# Enhancing research integrity and data quality through standardized electronic case report forms

**DOI:** 10.36416/1806-3756/e20240292

**Published:** 2024-09-16

**Authors:** Fabia Diniz-Silva, Juliana Carvalho Ferreira

**Affiliations:** 1. Divisao de Pneumologia, Instituto do Coracao, Hospital das Clinicas, Faculdade de Medicina, Universidade de Sao Paulo, São Paulo (SP) Brasil.; 2. Methods in Epidemiologic, Clinical, and Operations Research-MECOR-program, American Thoracic Society/Asociación Latinoamericana del Tórax, Montevideo, Uruguay.

## PRACTICAL SCENARIO

The Brazilian Research in Intensive Care Network (BRICNet) designed a multicenter, multinational, cohort study with a 28-day follow-up that will include patients with acute respiratory failure, in transition to spontaneous ventilation in intensive care units in Latin America.^1^ Given the multicentric nature of the study and the need to collect data in several countries, which also speak different languages, they were concerned that collecting data in paper forms could lead to errors and opted for using an electronic case report form (CRF) on the Research Electronic Data Capture (REDCap) platform.^2^

## DATA COLLECTION

Data collection is a crucial step in the scientific research process, as the quality of the data obtained directly influences the validity of the results and the reliability of conclusions of the study. Researchers must be meticulous in selecting the appropriate method for data collection to ensure that the collected data effectively address the research question.

The use of CRFs ensures standardization in obtaining information and allows for systematic data collection, minimizing variability and bias that may arise from non-standardized collection methods. CRFs should be well-structured, easy to complete, and designed to collect high-quality data. Only the minimum amount of data necessary to address the research question should be collected, avoiding the inclusion of excessive or irrelevant information.

A significant advancement in data collection has been the use of electronic forms instead of paper forms, as data collected on paper forms must be transferred to an electronic database to perform data analysis, and data entry errors can occur.^3^ The use of electronic forms significantly improves data quality control, as it verifies invalid responses and missing data in real time (Chart 1). Electronic CRFs serve as an interface that feeds data into a database and avoid the need to type data directly into a spreadsheet, which is prone to mistakes and should not be an option in research studies. When researchers type data directly into a spreadsheet, they can accidentally type data into the wrong column or line, type over data, and mistakes can go unnoticed. There are several methods for collecting data in electronic forms that directly feed a database, including some that are free of charge and used for informal surveys. However, in order to be reliable for collecting data in research studies, electronic forms need to be built to ensure the integrity of the data, protecting the database from unintended data changes or deletions, and protecting the confidentiality of patient data. 

**Chart 1:** Comparison of paper and electronic data collection forms.

Aspect	Paper data collection forms	Electronic data collection forms
Implementation	Simple to implement without the need for technological infrastructure	Needs access to technological resources
Initial cost	Generally lower, as it does not require electronic equipment	May be more expensive initially due to the need for electronic devices (computers, tablets, mobiles)
Accessibility	Can be used in locations with limited or no access to electronic devices or the Internet.	Allows easy sharing and remote completion via Internet-connected devices
Human Error in Transcription	Increased risk of errors when transcribing data into an electronic database	Minimizes transcription errors by allowing direct entry of data in electronic format
Collection and Processing Time	The process of collecting and subsequently digitizing data is time-consuming and can delay data analysis	Faster data collection and processing, with the possibility of direct export for statistical analysis
Quality Control	Difficulty in applying automatic quality controls; manual review is required	Better quality control with real-time data validation, alerting to missing or invalid data
Security and Confidentiality	Paper-based data is more vulnerable to losses, physical damage, and unauthorized access	Increased security with data encryption, access control, and automatic backups, complying with data protection regulations
Scalability	Difficult to scale for large studies, as it requires physical storage of large volumes of paper	Easily scalable for studies of any size, with digital storage and database expansion capabilities

## REDCAP

The REDCap software,^2^ developed by Vanderbilt University, is a web-based platform designed to facilitate the development of electronic data capture forms to be used in research. With REDCap, users can create customized data collection forms and questionnaires according to the specific needs of their studies or download instruments from a shared online library, saving time and resources.

To ensure the quality and integrity of the data collected, REDCap has a series of functionalities that assist data verification in real time. The platform allows the configuration of validation rules that automatically check data consistency and alert users in cases of missing or invalid data, such as text response in a numeric field or values out of range. Additionally, REDCap includes the branching logic feature, which automatically displays only the relevant questions based on previous responses. For example, if data being entered corresponds to a male participant, the question regarding pregnancy status will not appear. This ensures that only pertinent questions are presented, enhancing the respondent’s experience and minimizing the collection of irrelevant data.

Another important feature of REDCap is that different access permission levels can be defined for team members, ensuring that only authorized personnel have access to sensitive information collected, maintaining data confidentiality.

REDCap provides a multi-language feature in which the language of the form is displayed in the language of the person collecting the data but feed the same database. In the study described in our practical scenario, the investigators translated the forms from Portuguese to Spanish, and users collecting data in each of the 41 centers chose which language they preferred; data from centers in Peru and Brazil, for example, are saved on a single database for posterior data analysis.

Electronic CRF platforms such as REDCap should be relatively easy to use and feature an intuitive interface that allows users to set up their studies quickly and efficiently.

## KEY MESSAGES

- High-quality data collection is essential for the validity and reliability of research, and using standardized CRFs minimizes variability and bias.
- Electronic forms, particularly platforms like REDCap, enhance data quality by enabling real-time data verification, reducing errors associated with manual data entry, and ensuring data integrity and confidentiality.
- REDCap offers customizable data collection forms, validation rules, branching logic, multi-language support, and differential access permission levels, making it a powerful tool for research data management.
